# Anti-fibrillation and trans-fibrillation activities of L-arginine modified magnetic nanoparticles on lysozyme

**DOI:** 10.1016/j.bbrep.2026.102551

**Published:** 2026-03-23

**Authors:** Vahid Alimohammadi, Fatemeh Eshari, Faezeh Kashanian, Ali Akbar Moosavi-Movahedi, Seyd Ali Seyed-Ebrahimi, Mehran Habibi-Rezaei

**Affiliations:** a School of Biology, College of Science, University of Tehran, Tehran, Iran; b Advanced Magnetic Materials Research Center, School of Metallurgy and Materials Engineering, College of Engineering, University of Tehran, Tehran, Iran; c Institute of Biochemistry and Biophysics (IBB), University of Tehran, Tehran, Iran

**Keywords:** Hen egg white lysozyme (HEWL), Magnetic nanoparticles (MNP), L-Arginine modified magnetic nanoparticles (RMNP), HEWL amyloid aggregate (HAA), Anti-fibrillation, Trans-fibrillation

## Abstract

Over the last two decades, the application of nanoparticles (NPs) in the investigation of protein and peptide aggregation and amyloid-related diseases has been extensively studied. Here, we investigate anti-fibrillation and *trans*-fibrillation activities of L-Arginine modified Magnetic NPs (RMNPs, Fe_3_O_4_@Arg) on hen egg white lysozyme (HEWL) as a model protein and HEWL amyloid aggregate (HAA, i.e., aggregated HEWL fibrils). RMNPs were prepared by the co-precipitation method and subsequently characterized using X-ray diffraction (XRD), vibrating sample magnetometry (VSM), Fourier transform infrared spectroscopy (FT-IR), and scanning electron microscopy (SEM). The comparison was made among Arg, Magnetic NPs (MNPs), and RMNPs to explore their anti-fibrillation activities on HEWL. Using intrinsic tryptophan (Trp) and extrinsic thioflavin T (ThT) fluorescence assays, circular dichroism (CD) spectroscopy, and electron microscopy methods, for instance, we present the anti-fibrillation and *trans*-fibrillation activities of the RMNPs on HEWLs or HAAs. Accordingly, fibrillation was inhibited more than 80 percent, and fibrils were longitudinally rearranged to form late amyloid aggregates with a 57< nm diameter from early aggregates with a <14 nm diameter, respectively. The ability to inhibit HEWL amyloid formation highlights the concentration-dependent effects of the investigated NPs on HEWL at different stoichiometric ratios of MNPs and RMNPs. Moreover, RMNPs can interact with HEWL amyloids *in vitro* and alter the structure of the aggregates. Collectively, our findings shed light on the anti-fibrillation and *trans*-fibrillation effects of RMNPs on HEWL and HEWL amyloid aggregate (HAA), respectively. In this study, we aim to investigate the potential preventive effects of RMNPs on protein structural *trans*-formation and fibrillation, as well as their potential to promote the *trans*-fibrillation of resulting fibrils, representing a novel strategy to interfere with protein aggregation-related disorders.

## Highlights

• Distinctive effects of Arg, MNP, and RMNP on the morph aggregation of HEWL and HAA are reported.
• RMNP stabilizes the native form of HEWL, which is inversely correlated with increasing NP: protein ratios.
• The anti-fibrillation effect of MNPs is further improved upon incorporating Arg in the MNP as RMNP.
• RMNP shows trans-fibrillation effect on the fibrillated HEWLs as HAA. This effect is correlated with the RMNP:HAA ratios.

## Graphical abstract

**Figure:**
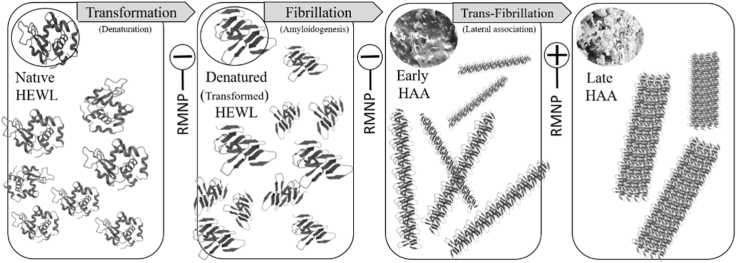

## Abbreviations:

Aβ42: Amyloid β 42
Arg: L-Arginine
BSA: Bovine serum albumin
CD: Circular Dichroism
FTIR: Fourier Transform Infrared spectroscopy
HAA: HEWL Amyloid Aggregate
HEWL: Hen egg white lysozyme
hIAPP: Human islet amyloid polypeptide
MNPs: Magnetic (Fe_3_O_4_) Nanoparticles
NPs: Nanoparticles
RMNPs: L-Arginine modified Magnetic [Fe_3_O_4_@Arg] Nanoparticles
SEM: Scanning Electron Microscopy
α-Syn: α-Synuclein
ThT: Thioflavin T fluorescence assay
Trp: Tryptophan
VSM: Vibrating Sample Magnetometry
XRD: X-ray Diffraction

## 1 Introduction

Proteins spontaneously fold into their functional native conformation with the lowest Gibbs free energy state in a non-protected or chaperon-protected environment [1,2]. However, protein aggregates can interfere with the folding pathway and may form during the unfolding or refolding processes [3,4]. Protein aggregation is a phenomenon in which protein conformational intermediates combine to form unordered amorphous or ordered fibrillary amyloid aggregates. This process leads to complications in the production of biotechnology products, such as enzymes and biopharmaceuticals, and is also associated with conformational diseases like Alzheimer's disease (AD), Parkinson's disease (PD), and Huntington's disease (HD), which are known as amyloid diseases [5]. As a brief note, the common feature of amyloid diseases is the transformation of proteins from a soluble form into insoluble β-sheet-rich amyloid aggregates. Accumulation of these aggregates potentially has toxic consequences, leading to cellular destruction by disrupting cell homeostasis and deregulating intracellular transport and membrane integrity [6,7]. Amyloid aggregate toxicity is involved in a variety of chronic diseases, most notably AD, which accounts for approximately 70% of dementias worldwide [8,9]. Recently, multiple review articles have detailed the list of proteins whose aggregation and/or fibrillogenesis have been reported to be inhibited by nanoparticles (NPs). Wang S. et al. have provided the inhibitory effect of various types of nanoparticles on the representative aggregation-prone peptides or proteins, including Aβ42, α-Syn, insulin, BSA and HEWL [10]. The list comprises metallic (e.g. zinc oxide, gold, silver and iron oxide); carbon-based NPs (e.g. Fullerene, carbon nanotube, graphene and graphene oxide); silica-based NPs (e.g. silicon, silica); polymer-based NPs (e.g. chitosan, poly lactic-co-glycolic acid, poly(trehalose). Hen egg white lysozyme (HEWL) is a single-subunit glycoprotein consisting of 129 amino acid residues with a molecular mass of 14.3 kDa and hydrolyzing activity (EC 3.2.1.17) [11]. It has emerged as a prototypical amyloidogenic protein for investigations on protein structure and function, and it is one of the most prevalent proteins commonly used in the study of model amyloid aggregation due to its well-established three-dimensional structure, availability, folding-unfolding mechanism, and conformational stability [12]. Numerous strategies have been explored to improve the stability of native protein structures under deteriorating conditions, thereby preventing unwanted protein aggregation [13,14].

Nanoparticles (NPs) have become increasingly popular in recent years due to their wide range of applications in areas such as diagnosis, analysis, and therapy [15]. NPs are produced from various materials with different physicochemical properties, which can affect protein structure and function [[16], [17], [18]]. Among them, magnetic nanoparticles (MNPs) possess a unique superparamagnetic property that can be used to control the transition of amyloid fibril dispersions [19]. Additionally, among 15 examined amino acids, L-Arginine (Arg; R) has revealed the best results for anti-fibrillation activities [20,21]. However, the prominent properties of MNPs and Arg have already been explored, and the interference of Arg-modified magnetic NPs (RMNPs, Fe_3_O_4_@Arg) on protein aggregation remains to be explored. Despite these advances, the specific effects of L-arginine–modified magnetic nanoparticles (Fe_3_O_4_@Arg) on HEWL fibrillation and on the structural remodeling of preformed HEWL amyloid aggregates remain insufficiently understood. Therefore, in this study, we investigate the concentration-dependent anti-fibrillation and *trans*-fibrillation activities of RMNPs on HEWL and HEWL amyloid aggregates (HAA), placing our findings within the broader mechanistic framework of nanoparticle-mediated modulation of amyloid pathways. Therefore, the current report aims to study the interference of MNPs or RMNPs on HEWL and HEWL amyloid aggregates (HAA, i.e., aggregated HEWL fibrils).

## 2 Materials and methods

### 2.1 Materials

Iron (II) Chloride Tetrahydrate (FeCl_2_.4H_2_O) and Iron (III) Chloride Hexahydrate (FeCl_3_.6H_2_O) purchased from Sigma-Aldrich were used as the precursors. HEWL, as a model protein, was also obtained from Sigma-Aldrich. Thioflavin T (ThT) was purchased from Fluka. Arg and other chemicals were from Merck (Darmstadt, Germany) unless otherwise stated. To monitor the fluorescence intensities, we used a BioTek, Synergy™ H4 Hybrid microplate reader. All experiments were performed in triplicate unless otherwise stated.

### 2.2 Preparation and characterization of MNPs and RMNPs

**MNP Synthesis-** MNPs were synthesized by the co-precipitation method under a nitrogen atmosphere as described in previous studies [22,23]. Briefly, FeCl_3_.6H_2_O and FeCl_2_.4H_2_O salts were mixed in a ratio of 2:1 (M) and dissolved in 20 ml of deionized water. The mixture was stirred at 50 °C for 30 min, and ammonia solution was added by a syringe to prepare MNPs after 30 min of stirring. Finally, the MNPs were washed with ethanol, then with deionized water three times, separated from the liquid phase by a permanent magnet, and dried at room temperature.

**RMNP Synthesis-** For RMNPs synthesis, FeCl_3_.6H_2_O and FeCl_2_.4H_2_O salts in a ratio of 2:1 (M) were dissolved in 20 mL of deionized water, stirred at 300 rpm at 50 °C for 30 min. While stirring, a 10 μM Arg solution in deionized water was added dropwise, followed by adding 7 mL ammonia (25 wt%) by syringe. RMNPs were separated from the liquid phase using a permanent magnet, washed with ethanol followed by deionized water thrice to eliminate impurities, and finally dried at room temperature.

**NPs Analysis-** X-ray diffraction (XRD) patterns of MNPs and RMNPs were determined by a Regiko instrument with a scan range of 5 ° <2θ < 90 °. Measurement of the sample magnetization was carried out using vibrating sample magnetometry (VSM) (Meghnatis Daghigh Kavir Co.; Kashan Kavir; Iran) at room temperature. A CamScan MV2300 Scanning Electron Microscopy (SEM) was used to observe the NPs and the amyloids formed after interaction with the NPs. The zeta potential of NPs was measured by Malvern ZS-Nano series 1001767, and Fourier transform infrared (FT-IR) spectra were recorded by a Magna-Nicolet 550 spectrometer in KBr pellets.

### 2.3 Lysozyme amyloid aggregation

HAA was prepared by incubating the soluble protein (0.147 mg mL^−1^) in 70 mM glycine, pH 2.2, at 54^°^C, and stirring at 205 rpm for 24 h. The formation of amyloid aggregates was monitored by characteristic changes in ThT extrinsic fluorescence intensity using a spectrofluorimeter. To investigate the anti-fibrillation effects of NPs, HEWL (0.147 mg mL^−1^) was treated with NPs: HEWL ratios of 0:1 (as control), 1:1, 3:1, and 5:1 by adding NPs from a 5 mg mL^−1^ stock in four separate tubes. Three different concentrations of NPs were used: 0.147, 0.441, and 0.735 mg mL^−1^, and the control sample was prepared from a protein solution with a concentration of 0.147 mg mL^−1^ without NPs. To explore the *trans*-fibrillation effect of RMNPs, HAA was used after washing instead of HEWL. Samples without (as a control) or with NPs (similar to the anti-fibrillation process) were prepared, and incubation was performed as described before.

### 2.4 Fluorescence spectroscopy

Intrinsic tryptophan (Trp) fluorescence emission spectra of HEWL in the absence (as control) or presence of different concentrations of RMNPs were recorded in a 96-well quartz plate at room temperature after excitation at 280 nm. The fluorescence spectra were taken in the wavelength range of 300‒400 nm at 25 °C. ThT**,** a benzothiazole fluorescent dye, shows a remarkable fluorescence intensity increase upon binding to β-sheet-rich amyloid aggregates, enabling both quantification of amyloid formation and monitoring of aggregation kinetics [24]. The fibrillary state of incubated HEWL was determined by extrinsic ThT fluorescence, where ThT at 250 μM was prepared in 20 mM sodium phosphate buffer with pH 7.4. Then, 0.003 ml of ThT solution was added to 0.3 ml of each sample, followed by pre-incubation for 15 min at room temperature in the dark. Fluorescence assessment was carried out by emission scanning in the wavelength range of 440 – 660 nm after excitation at 420 nm. To study the kinetics of the *trans*-fibrillation process, we monitored the temporal evolution of the ThT fluorescence intensity of HEWL in the absence or presence of RMNPs that were incubated at 27 °C, under stirring at 205 rpm for up to 24 h. All data are the average of three experiments.

### 2.5 Circular dichroism spectroscopy

To investigate the effect of NPs on the secondary structure of HEWL at 0.147 mg mL^─1^, circular dichroism (CD) spectroscopy (AVIV circular dichroism spectrometer Model 215) was used in the absence or presence of different concentrations of NPs after 24-h incubation, as mentioned before. The far-UV region was scanned in the wavelength range of 190 - 250 nm, and the average of five consecutive scans was corrected using the buffer as a blank. The CD data were expressed in terms of the mean residual ellipticity in deg.cm^2^.dmol^─1^, and the CDNN software was used for deconvolution of the far-CD spectra to measure secondary structure changes and percentage composition of the different secondary structural elements.

### 2.6 Scanning electron microscopy (SEM)

The resulting aggregates in the presence or absence of RMNPs were observed by SEM; the sample solution was diluted 100-fold with pure water. Subsequently, 1 μL of the diluted samples was placed on a silicon and air-dried for 10 min, then the images were prepared using SEM; CamScan MV2300, and fibril width values were analyzed using ImageJ bundled with 64-bit Java 1.8.0_112 software.

## 3 Results & discussion

Recently, the application of nanoparticles (NPs) in bioindustrial and biomedical fields has raised safety concerns regarding toxicity and environmental impact [23,25]. Among these, magnetic nanoparticles (MNPs)—such as L-arginine-modified variants —have garnered substantial interest for drug delivery, imaging, and hyperthermia applications despite these limitations [25]. In this study, we decorated MNPs with Arg to explore the anti-fibrillation (inhibition of fibrillogenesis) and *trans*-fibrillation (fibrils compact side-by-side) activities of RMNPs on HEWL as a model protein.

### 3.1 MNPs and RMNPs characterization

**Fig. 1:**
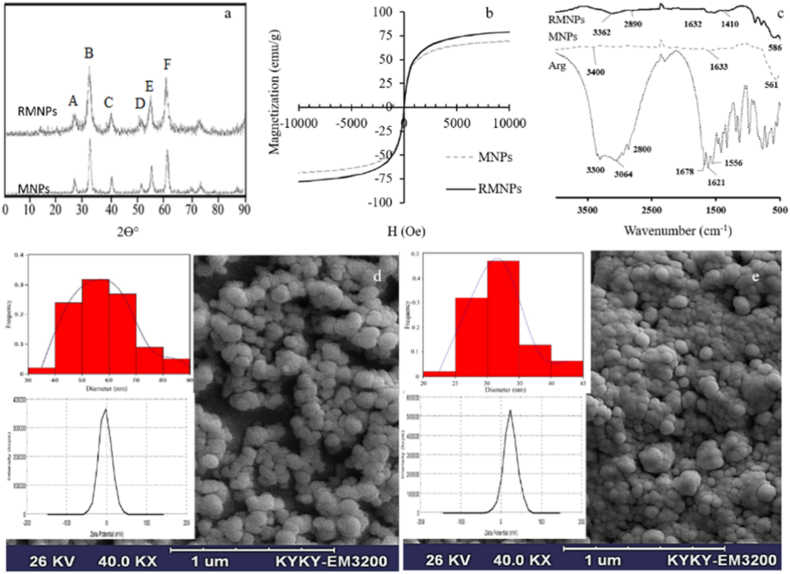
Characterization of MNPs and RMNPs using XRD (a) VSM (b) and FT-IR(c). SEM images of the MNPs (d) and RMNPs (e) are shown including the particle size distribution analysis using ImageJ (the upper insets), and related Zeta potential of the NPs using DLS (the lower insets).

MNPs and RMNPs were synthesized and characterized by XRD, VSM, and FT-IR analyses. Moreover, SEM images of MNPs and RMNPs were acquired, and particle size analysis was conducted using ImageJ software (Fig. 1). The XRD patterns of the nanoparticles exhibited characteristic diffraction peaks indexed as (A, B, C, D, E, and F), corresponding to the (220), (311), (400), (422), (511), and (440) crystallographic planes, respectively (Fig. 1a). These six main peaks matched well with the standard reference data from the International Center for Diffraction Data (JCPDS Card: 19-629). Magnetization measurements performed by VSM demonstrated that both MNPs and RMNPs exhibit superparamagnetic behavior, characterized by the absence of a hysteresis loop and negligible coercivity (Fig. 1b). The saturation magnetization values were approximately 81 and 69.9 emu·g^−1^ for MNPs and RMNPs, respectively. FT-IR spectra (Fig. 1c) provided direct evidence of successful nanoparticle synthesis and functionalization. The Fe–O bond absorption peak was observed around 561 cm^−1^ for both MNPs and RMNPs. For Arg, characteristic bands included a broad N–H stretching vibration between 3000 and 3300 cm^−1^ and a strong N–H bending vibration near 1550–1640 cm^−1^. In RMNPs, absorption peaks in the 1000–1300 cm^−1^ range were attributed to C–N stretching vibrations, while N–H bending and C–H stretching vibrations appeared at 1630 and 2970 cm^−1^, respectively. Additionally, the N–H stretching vibration of Arg overlapped with the O–H stretching band of magnetic nanoparticles around 3000–3400 cm^−1^. Collectively, these observations confirm the successful surface decoration of RMNPs with Arg.

The average particle size was estimated to be approximately 56.0 ± 1.6 nm for MNPs (Figs. 1d) and 33.0 ± 2.6 nm for RMNPs (Fig. 1e). Additionally, the surface charge of the nanoparticles was evaluated by measuring the zeta potential using a zeta sizer in glycine buffer. MNPs exhibited a slight negative surface charge (∼−3.57 mV), whereas RMNPs displayed a positive surface charge of approximately +23.1 mV.

### 3.2 Effects of MNPs and RMNPs on HEWL (anti-fibrillation effect)

**Fig. 2:**
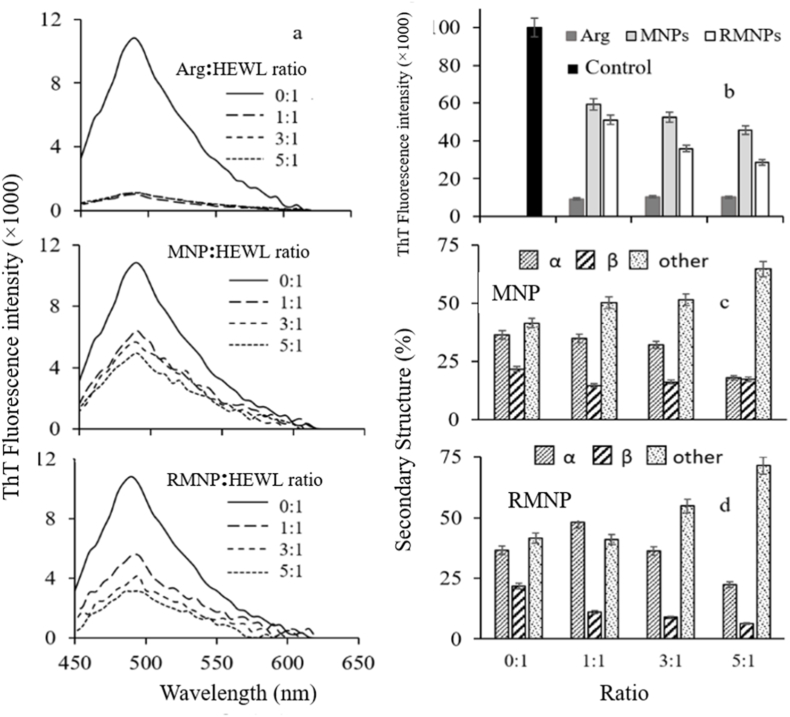
Comparative analysis of the anti-fibrillation effect of Arg, MNPs, and RMNPs on 0.147 mg mL^─1^ HEWL under amyloidogenesis conditions (at 54 °C, pH 2.2 and 205 rpm over 24 h). The fluorescence spectra (a), the normalized extrinsic ThT fluorescence intensities of HEWL in the absence (as control) or presence of Arg or NPs (b), and the secondary structures of incubation products as determined by CD spectroscopy at three representative ratios as MNPs: HEWL (c) or RMNPs:HEWL (d).

The anti-fibrillation effects of Arg, MNPs, and RMNPs on HEWL were evaluated using extrinsic thioflavin T (ThT) fluorescence, circular dichroism (CD), and intrinsic tryptophan (Trp) fluorescence analyses (Fig. 2). ThT fluorescence assays quantified HAA as the fibrillar aggregates formed during HEWL incubation. Increasing concentrations of additives led to a reduction in β-sheet content in samples incubated with varying concentrations of Arg or each of the nanoparticles (MNPs or RMNPs) for 24 h. As shown in Fig. 2a and b, ThT fluorescence intensity decreased in a concentration-dependent manner with increasing Arg levels, indicating effective suppression of protein aggregation at all tested ratios, consistent with previous studies [26,27]. Similarly, both MNPs and RMNPs demonstrated concentration-dependent anti-fibrillation activity, with RMNPs exhibiting greater inhibitory effects than MNPs. Fig. 2b presents the mean relative ThT fluorescence intensities ± standard deviation (SD) as error bars. Fluorescence observations are supported by the effect of MNPs and RMNPs on HEWL secondary structure in the absence or presence of three representative MNPs:HEWL and RMNPs:HEWL ratios, using CD analysis as shown in Fig. 2c and d, respectively. While MNPs caused a slight reduction in β-sheet content, RMNPs induced a substantially greater, ratio-dependent decrease in β-sheet formation. Additionally, RMNPs significantly increased α-helical content at the 1:1 ratio. In both treatments, other secondary structure components besides α-helices were also altered, suggesting notable conformational changes in HEWL induced by increasing nanoparticle concentrations. In terms of anti-fibrillation activity, an obvious decreasing trend was observed, which was higher for the Arg than RMNPs and MNPs. This indicates an improved anti-fibrillation effect for RMNPs after MNPs surface decoration with Arg. It is worth mentioning that concentration dependency in the RMNPs: HEWL ratios of 1:1, 3:1, and 5:1 plays the most prominent role.

Six tryptophan residues at positions 28, 62, 63, 108, 111, and 123 contribute to the intrinsic fluorescence of HEWL. Trp62 and Trp108, proximal to the substrate-binding site, primarily drive fluorescence emission. During denaturation, HEWL exhibits reduced intrinsic fluorescence due to altered local environments of these Trp residues [28,29].

**Fig. 3:**
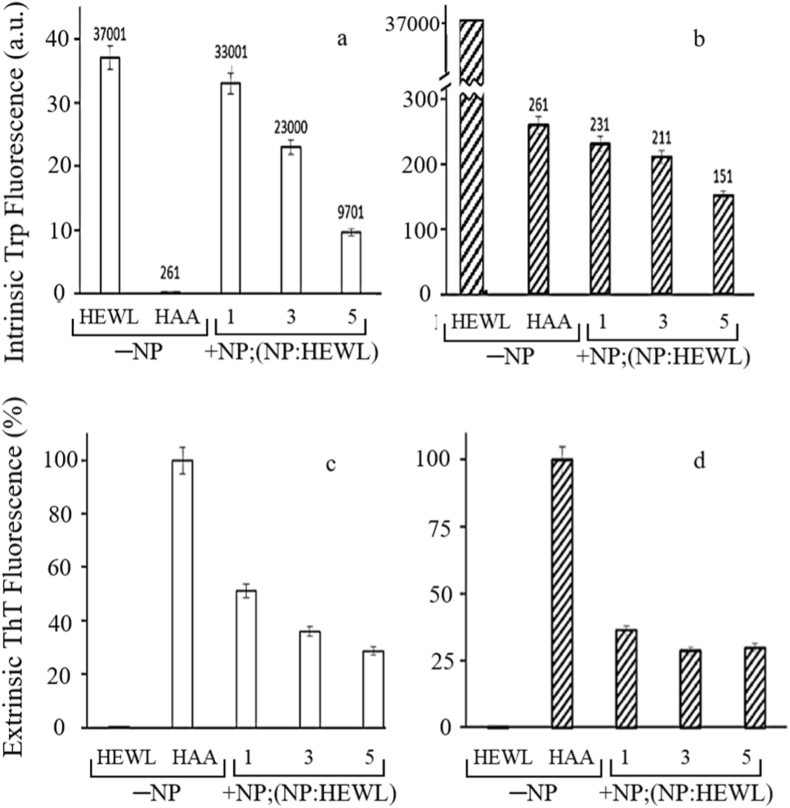
Exploring of the anti-fibrillation and *trans*-fibrillation effects of RMNPs on HEWL(a) and HAA (b) using Trp intrinsic fluorescence assay and ThT extrinsic fluorescence assays for anti-fibrillation and *trans*-fibrillation activities on HEWL(c) and HAA (d) for 0:1, 1:1, 3:1, and 5:1 ratios of RMNPs: HEWL or HAA (equal to 1, 3 and 5 in the chart). The data are the mean of three repeats in each case.

As depicted in Fig. 3, the fluorescence intensity significantly decreases upon HEWL fibrillation toward the formation of HAA in the absence of RMNPs. This is explained by substantial protein structural alteration under an unprotected fibrillation process. However, RMNPs effectively preserved the protein conformation to keep fluorescence intensity closer to the native state at the lowest examined ratio (1:1) than at the highest examined ratios (3:1) and (5:1) (Fig. 3a). The latter observation is well in agreement with the reported concentration-dependent effect of NPs, which is inversely proportional with protein structural preservation or perturbation at lower and higher concentrations, respectively [27]. More interestingly, when the HEWL amyloid aggregate was treated by RMNP ratios after washing, further decreases in intrinsic Trp fluorescence emission were observed. This is assumed to be due to structural alteration toward aggregate compactness and further fluorophore quenching in the presence of RMNPs. The observed decreases were proportional to the increase in the RMNP ratio (Fig. 3b).

Although ThT can bind to HAA and emit fluorescence, soluble HEWL as a negative control shows almost no ThT fluorescence emission (Fig. 3c). The product of HEWL fibrillation in the presence of RMNPs exhibits reduced fluorescence emission correlated with the RMNPs:HEWL ratio. These observations can be attributed to the combined anti-fibrillation and *trans*-fibrillation effects of the nanoparticles, with the *trans*-fibrillation effect becoming dominant at higher ratios. In other words, RMNPs primarily exhibit anti-fibrillation activity at lower concentrations, shifting towards dominant *trans*-fibrillation effects at higher ratios. Continuing, the ThT fluorescence emission of washed HAA (product of HEWL fibrillation in the absence of RMNP) treated with varying RMNP ratios was examined (Fig. 3d). Accordingly, ThT extrinsic fluorescence of HAA decreased as the RMNP ratio increased, likely reflecting the *trans*-fibrillation effect of RMNPs on HAA**.**

### 3.3 Effects of RMNPs on HAA (*trans*-fibrillation)

**Fig. 4:**
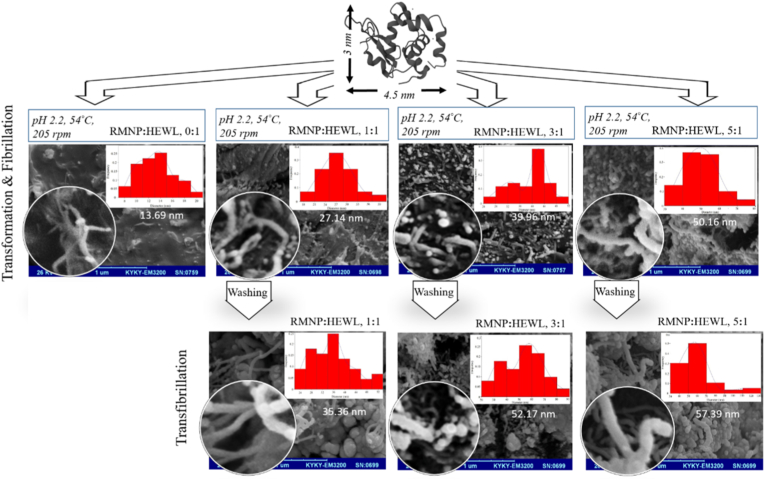
a) SEM images showing the anti-fibrillation and *trans*-fibrillation activities of RMNP at different ratios: 0:1 (0A), 1:1 (1A), 3:1 (3A), and 5:1(5A). Upper row displays fibrillation of HEWL to HAA under conditions of pH 2.2, 54 °C and 205 rpm, illustrating the diameters of the fibrils formed. The lower row shows further *trans*-fibrillation of washed HAA in the presence of RMNP ratios at 1:1 (1T), 3:1 (3T), and 5:1(5T).

Fig. 4 presents the scanning electron micrographs of the products of HEWL transformation to the denatured conformations induced by harsh conditions (pH 2.5, 54 °C, 205 rpm) which make them fibrillation-prone to generate HAA (inset to figures are represent size distributions). The samples were incubated in the absence or the presence of representative ratios of RMNPs as 0:1, 1:1, 3:1, and 5:1 ratios, respectively. Accordingly, the diameter of the fibrils increased in the presence of RMNPs, and such an increase was again correlated with increasing the ratios. As depicted, HEWL fibrillation ordinarily takes place in the absence of RMNP to develop 13.69 nm diameter HAA fibrils (HAA-0). In addition to the revealed anti-fibrillation effects of the RMNP on HEWL presented in Fig. 2, Fig. 3, the fibrillation products as early HAA are subjected to the promoted *trans*-fibrillation effect of RMNPs at three representative ratios, to bring about the side-by-side arrangement of detained early HAA fibrils to result in higher diameters at 27.14 nm, 39.96 nm, and 50.16 nm proportional to the increasing RMNPs:HEWL ratios (1:1, 3:1, and 5:1), respectively. However, more effective *trans*-fibrillation effects were observed when HAAs (instead of HEWL) were incubated after washing with the same ratios of RMNPs:HAA to generate late HAA characterized with the higher diameters at 35.36 nm, 52.17 nm, and 57.39 nm, respectively (Fig. 4). While SEM involves drying artifacts, the consistent ratio-dependent morphological transition across all samples suggests that the observed thickening reflects genuine solution-phase rearrangement.

Based on the evidence, fibril diameter is expected to adopt a narrow range of values. *Close* et al. reported that fibril morphologies formed by an amyloidogenic immunoglobulin light chain (AL1) using cryo-electron microscopy (cryo-EM) vary with the fibril diameter, ranging from 6 to 22 nm [30]. However, they did not consider side-by-side interactions along the fibril axis and acknowledged that such an assumption may break down progressively as the fibril width increases [30]. As shown in Fig. 4, regardless of whether we start with HEWL or HAA, the fibril diameter increases with increasing RMNPs in the ratio. *Naik* et al. reported widely dispersed HEWL-amyloid fibrils and scattered particles upon its treatment with super-paramagnetic magnetic nanoparticles (SPION) [31]. Similar observation has also been reported in the case of transferrin [32]. Similarly, gold NPs (AuNPs) have affected insulin fibrillation to make them much shorter and more compact [33]. Accordingly, RMNPs along with their anti-fibrillation effect as presented in Fig. 3, make generated fibrils even though under inhibited conditions, to be *trans*-fibrillated, which results in increasing fibril diameters.

**Fig. 5:**
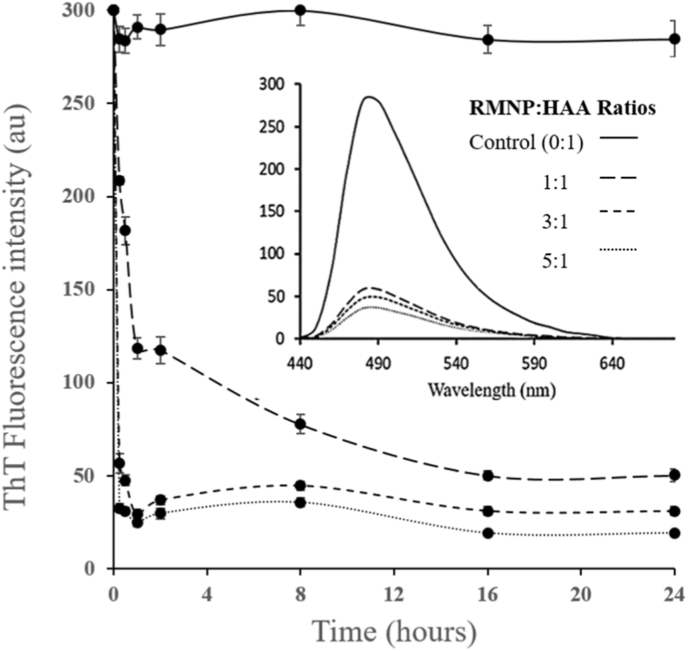
Time course of ThT fluorescence changes of HAA after washing as a function of time over 24 h in the presence of different RMNPs: HAA ratios at 0:1 (control), 1:1, 3:1, and 5:1). The inset shows the ThT fluorescence emission spectra after 24 h.

To provide a clear picture of the *trans*-fibrillation activity of RMNPs on HAA, a time-dependent ThT extrinsic fluorescence was monitored in the absence (as a control) and presence of RMNP ratios. As depicted in Fig. 5, the kinetics of the *trans*-fibrillation activity of RMNPs at four RMNP:HAA ratios of 0:1 (as control), 1:1, 3:1 and 5:1 were measured using the ThT fluorescence assay. The ThT fluorescence assay was performed at 8-time intervals over 24 h. Accordingly, the ThT fluorescence emission decreased with increasing RMNP concentration, likely reflecting the concentration-dependent *trans*-fibrillation effect of RMNPs on HAA. The inset of Fig. 5 shows the decreasing ThT fluorescence emission spectra in different RMNPs:HAA ratios (1:1, 3:1, and 5:1) after 24 h. It is important to emphasize that reduced ThT fluorescence does not necessarily imply the absence of fibrillary structures, but rather reflects altered β-sheet accessibility or packing density upon the lateral association process. SEM analysis confirms the persistence of fibrillar morphology despite reduced ThT intensity, supporting a remodeling rather than dissolution mechanism.

Limiting protein aggregation offers substantial benefits in biology and medicine by preventing consequences associated with a wide variety of protein aggregation-related diseases. Furthermore, soluble protofibrils, as highly toxic early amyloid intermediates, exert greater cytotoxicity than mature fibrils as late aggregates; thus, *trans*-fibrillation into insoluble late-stage fibrils mitigates their detrimental impact [34]. RMNPs are small in size, yet possess a high surface area to volume ratio, which makes them prone to interact with HEWLs to stabilize them at low RMNP concentrations, probably through improving HEWL hydration. However, RMNPs at high concentrations not only do not play as stabilizers or anti-fibrillation agents but also act as *trans*-fibrillation agents, probably through reducing the protein hydration layer, making the condition more suitable for longitudinal interactions between fibrils to increase the diameter.

As a result, both MNPs and RMNPs can interact with amyloid fibrils *in vitro*, resulting in self-assembled hybrid longitudinal aggregates. It has been reported that MNPs can adsorb onto amyloid fibrils and change their morphology [35,36]. Accordingly, we hypothesize that increasing the RMNPs:HEWL ratio alters HAA morphology, enhancing its propensity for *trans*-fibrillation.

At low RMNP:HEWL ratios, HEWL forms a stabilizing corona on saturated RMNP surfaces, where Arg-mediated kosmotropic effects and adsorptive interactions stabilize the desired protein in its native conformation [22]. Arginine is widely used as a protein-stabilizing and aggregation-suppressing additive, in part due to its guanidinium group, which can engage in multiple hydrogen bonds, cation–π, and electrostatic interactions and can modulate the protein hydration shell [22]. By decorating MNPs with Arg as RMNP, we effectively combine the high surface-area and magnetic properties of Fe_3_O_4_ with the kosmotropic features of Arg to explicitly cite studies demonstrating Arg-mediated suppression of HEWL transformation while improving refolding yields and consequent fibrillation. Moreover, the increased positive zeta potential (+23.1 mV for RMNPs vs −3.57 mV for MNPs) is consistent with this enhanced interaction with HEWL (Section 3.1, 3.2). Despite this, residual denatured HEWL as aggregation prone species, undergoes hydrophobic-driven fibrillation, as shown here. Incorporation of the amino acid Arg in Fe_3_O_4_ (RMNP, Fe_3_O_4_@Arg) not only confers biocompatibility and non-toxicity while inhibiting lysozyme fibrillation [36], consistent with Arg's documented effects on other proteins [37,38]. Conversely, at high RMNP:HEWL ratios, exposed RMNP surfaces promote lateral fibril associations during *trans*-fibrillation, yielding thickened mature fibrils.

**Fig. 6:**
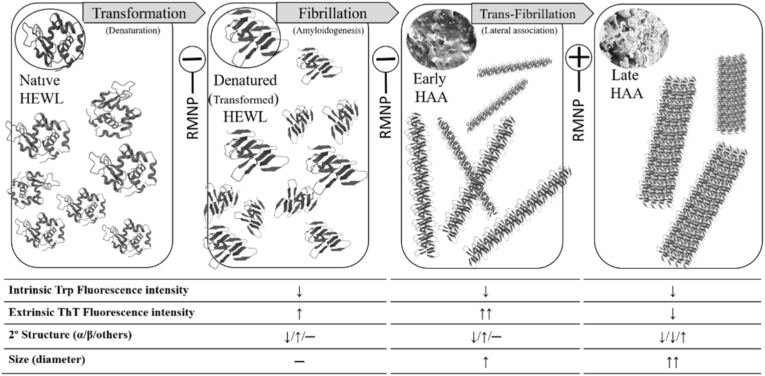
Schematic diagram illustrating RMNP interference in the HEWL structural transformation (denaturation) and aggregation at pH 2.2, 54 °C and 205 rpm for 24 h, to generate early HAA (protofibrillar intermediates) followed by their *trans*-fibrillation into thicker mature fibrils (late HAA). Bottom panel summarizes RMNP effects: increase (↑), decrease (↓), or no change (─) in intrinsic Trp fluorescence intensity, extrinsic ThT fluorescence intensity, secondary structure elements (α-helix, β-strand, others), and fibril diameter (size).

Given the worldwide prevalence of amyloid diseases, the anti-fibrillation and *trans*-fibrillation activities of RMNPs on the amyloid aggregation of HEWL have the potential to attract interest in various disciplines. Additionally, the simplicity of RMNP synthesis biocompatibility and biodegradability of these advanced nanomaterials make them ideal candidates for further research. In this study, we systematically examined the effect of varying concentrations of RMNPs on HEWL aggregation, as schematically illustrated in Fig. 6. The schematic depicts how RMNPs interfere with HEWL structural transformation (denaturation) and aggregation under harsh conditions (pH 2.2, 54 °C, and 205 rpm). Under these conditions, HEWL initially forms proto-fibrillar intermediates and early fibrillar aggregates (early HAA), which subsequently undergo *trans*-fibrillation to yield thicker, mature fibrils (late HAA). The lower panel of the schematic further summarizes the spectroscopic findings derived from experiments designed to elucidate the impact of RMNPs on the HEWL fibrillation pathway.

In recent years, increasing attention has been directed toward understanding how NPs influence fibrillogenesis pathways. It is well established that the size and physicochemical properties of NPs—including surface charge, hydrophobicity, and surface chemistry—are critical determinants of whether fibrillation is inhibited, redirected, or, in some cases, accelerated [39,40]. Mechanistic studies indicate that NPs can interfere with multiple stages of fibril formation. They may inhibit primary nucleation by binding to monomeric or early oligomeric species, forming a dynamic protein corona that removes these species from solution and thereby reduces the effective concentration available for fibril growth [41]. Additionally, nanoparticles can interact with partially formed aggregates, bind to exposed β-sheet regions, and hinder further peptide/protein recruitment [42]. Overall, current evidence suggests that NP–fibril interactions are highly context-dependent and governed by adsorption phenomena, electrostatic forces, hydrophobic interactions, and kinetic modulation. Here, we hypothesize trans-fibrillation as a potential mechanism underlying the inhibitory effect of RMNPs on HEWL fibrillation.

## 4 Conclusion

The results demonstrate that RMNPs have a significant impact on the structural changes of HEWL under harsh conditions, leading to anti-fibrillation followed by a process of *trans*-fibrillation. These findings were supported by comprehensive secondary structure analysis using CD spectropolarimetry, intrinsic tryptophan fluorescence, extrinsic ThT fluorescence data, and SEM imaging. Here, we demonstrate an inverse correlation between RMNP:HEWL ratios and the stabilizing effect of RMNPs on the native HEWL conformation. However, the study confirmed that RMNPs exhibit an anti-fibrillation effect in a concentration-dependent manner, similar to unmodified MNPs. However, due to the protein aggregation suppression property of arginine, the anti-fibrillation activity of RMNPs was found to be more effective than that of MNPs, regardless of their concentration. In conclusion, this work reports the interference of magnetic nanoparticles in the process of HEWL fibrillogenesis. The findings collectively shed light on the anti-fibrillation and *trans*-fibrillation effects of RMNPs on HEWL and HAA, respectively, as a possible mechanistic approach of the RMNP effect of HEWL Future studies are necessary to assess the therapeutic potential and potential risks associated with the *trans*-fibrillated structures, especially regarding protein folding disorders.

## Declaration

No content was generated or altered by AI; all scientific content, interpretations, and final revisions remain the sole responsibility of the authors. AI (Perplexity) was used exclusively for checking writing correctness for only the current revision.

## CRediT authorship contribution statement

**Vahid Alimohammadi:** Conceptualization, Investigation, Methodology, Writing – original draft. **Fatemeh Eshari:** Software, Writing – review & editing. **Faezeh Kashanian:** Conceptualization, Methodology, Validation. **Ali Akbar Moosavi-Movahedi:** Validation, Writing – review & editing. **Seyd Ali Seyed-Ebrahimi:** Methodology, Validation. **Mehran Habibi-Rezaei:** Conceptualization, Funding acquisition, Methodology, Project administration, Resources, Supervision, Validation, Visualization, Writing – review & editing.

## Declaration of competing interest

The authors declare no competing interests, financial or otherwise, that could have influenced the research presented in this manuscript.

## Data Availability

Data will be made available on request.
